# Subjective body image and recent weight-control attempts as independent correlates of adolescent mental-health problems: Analysis of the 2024 Korea Youth Risk Behavior Survey

**DOI:** 10.1192/j.eurpsy.2026.11148

**Published:** 2026-07-31

**Authors:** J. Kim, J. Jung, S. Kim

**Affiliations:** 1 Psychiatry, Daegu Catholic University School of Medicine, Daegu; 2 Fayston Christian School, Suji Campus, Yongin-si, Korea, Republic Of

## Abstract

**Introduction:**

Distorted body image and weight-control behaviour strongly relate to adolescent mental health. In cultures idealising thinness, perceiving oneself as overweight may affect wellbeing more than actual body mass.

**Objectives:**

This study examined independent and combined associations of subjective body-image perception and recent weight-control attempts with depression, anxiety, and suicidal ideation among Korean adolescents.

**Methods:**

Data were from the 2024 Korea Youth Risk Behavior Survey, a nationwide, web-based, school survey using a stratified multistage cluster design. For descriptive analysis, unweighted raw data were used (N = 54 653; male 51.4%, female 48.6%; metropolitan 43.0%, small/medium city 50.1%, rural 7.0%). Mental-health outcomes were self-reported depressive mood, clinically significant anxiety (GAD-7 ≥ 10), and suicidal thoughts during the past year. Main predictors were perception of being overweight and weight-control attempts within 30 days. Covariates included sex, grade, socioeconomic status, and body-mass index. Survey-weighted multivariable logistic regressions were conducted in R (v4.3.3) with adjusted odds ratios (aORs) and 95% confidence intervals (CIs).

**Results:**

Both perceived overweight and recent weight-control attempts were independently associated with poorer mental health after adjusting for BMI and covariates. For depression, perceived overweight (aOR = 1.21, 95% CI 1.11–1.31) and recent attempts (aOR = 1.48, 95% CI 1.38–1.59) increased risk, while BMI showed an inverse association (aOR = 0.97, 95% CI 0.96–0.98); interaction non-significant (p = 0.168). For anxiety, perceived overweight (aOR = 1.44, 95% CI 1.34–1.54) and attempts (aOR = 1.29, 95% CI 1.21–1.38) were significant, with a small negative interaction (aOR = 0.91, p = 0.032). For suicidal ideation, perceived overweight (aOR = 1.44, 95% CI 1.30–1.60) and attempts (aOR = 1.41, 95% CI 1.28–1.54) remained significant; interaction non-significant (p = 0.071). Across all models, subjective perception and behaviour showed additive, not multiplicative, effects on emotional distress.

**Conclusions:**

Perceiving oneself as overweight and attempting to lose weight independently predict depression, anxiety, and suicidal ideation among Korean adolescents, regardless of actual BMI. Subjective body image—not objective weight—was the stronger determinant of psychological vulnerability. These results suggest that prevention strategies should integrate body-image education and cognitive approaches promoting realistic self-perception, complementing physical-health programmes to improve mental wellbeing and reduce self-harm risk.

**Disclosure of Interest:**

None Declared

